# Health-Related Lifestyle Behavior and Religiosity among First-Generation Immigrants of Polish Origin in Germany

**DOI:** 10.3390/ijerph15112545

**Published:** 2018-11-13

**Authors:** Eva Morawa, Yesim Erim

**Affiliations:** Department of Psychosomatic Medicine and Psychotherapy, University Hospital of Erlangen, Friedrich-Alexander-University Erlangen-Nürnberg (FAU), 91054 Erlangen, Germany; yesim.erim@uk-erlangen.de

**Keywords:** immigrants, Polish, religiosity, lifestyle behavior, smoking, alcohol consumption, physical activity, overweight, obesity

## Abstract

*Background*: Health-related lifestyle behaviors such as smoking, alcohol consumption, physical inactivity and obesity are major cardiovascular risk factors. Previous studies have mostly demonstrated a favorable association between religiosity and these cardiovascular risk factors; however, no studies have investigated this relationship in Polish immigrants. The aim of this cross-sectional study was to examine the association between health-related lifestyle behaviors and religiosity in Polish immigrants in Germany. *Methods*: The smoking patterns, frequency of alcohol consumption, physical activity, and presence of overweight/obesity were assessed in 257 first-generation immigrants of Polish origin living in Germany. Religiosity was measured with the Centrality of Religiosity Scale (CRS, Huber, 2003) consisting of 15 items that categorized the respondents into intrinsically, extrinsically, and not/marginally religious. *Results*: After adjusting for various sociodemographic, migration, and health-related characteristics, intrinsic religiosity was significantly associated with a lower risk of being a smoker (odds ratios (OR) = 0.34, confidence intervals (CI) = 0.15–0.76) and was also associated with a lower risk of alcohol consumption (OR = 0.33, CI = 0.15–0.71), but a higher risk of being overweight/obese (OR = 2.53, CI = 1.15–5.56) in comparison with extrinsic/marginal religiosity. No significant relationship was found between religiosity and physical activity. *Conclusions*: In Polish immigrants, intrinsic religiosity acts as a protective factor against some cardiovascular risk factors (smoking and alcohol consumption).

## 1. Introduction

There has been increasing interest in examining the relationships between religious, spiritual, and health variables in medical and psychological research during the last decades. Despite scientific progress, the role of religion/religiosity is still substantial for a large proportion of humankind. According to the Global Index of Religion and Atheism from 2012 [1] (including 51,927 people from 57 countries in five continents), 59% of all people regard themselves as being religious. Religious beliefs and practices provide not only a sense of meaning in the lives of human beings but are also an important source of rules for (moral) behaviors.

### 1.1. The Religious Landscape in Poland and Germany

Religion plays an essential role in the lives of Poles. In Poland, 96% of the population declare their affiliation with the Roman Catholic Church [2], whereas only 28.5% of the citizens in Germany are Roman Catholics [3]. The high identification of the Poles with the Catholic faith as well as the religious, cultural, and political influence of the Catholic Church through the centuries until now are grounded in the history of Poland [4]. During times of foreign oppression, the Catholic Church contributed substantially to the preservation of the identity of the Polish nation, the Polish language, and traditions, as well as to the national resistance and fight for independence.

The statistics indicate not only the formal declaration of being Catholic of most Poles but also the high proportion of religious people in this nation. Eighty-one percent of Poles and 51% of the Germans describe themselves as religious [1]. According to the Centre for Public Opinion Research in Poland [5], 93% of all citizens in Poland consider themselves to be believers (85% believers and 8% strong believers). This trend of the Polish religiosity has remained stable over the last 20 years (between 92% and 97%). In 2016, on average, 50% of Catholics in Poland participated in the Sunday mass [5] compared to only 10.2% of Catholics in Germany [6].

In Germany, no statistics are available for the mass attendance of Polish immigrants. Many immigrants of Polish origin participate in Polish-speaking religious services in the “Polish Catholic Missions” in almost every large city in Germany. In these Polish pastoral centers, it is not only church services that take place but also religious education in Polish, Polish classes, and activities of various religious and cultural groups [4]. Therefore, the local Catholic Polish communities are places where the immigrants can maintain their indigenous language as well as religious and cultural traditions and thus preserve their identity [6].

### 1.2. Immigrants of Polish Origin in Germany

In Germany, more than every fifth person (22%) has a migration background, either having personally moved there or having at least one parent who moved [7]. There are approximately two million immigrants of Polish origin [8], one of the three largest immigration groups in German society today, the other two being Turkish and Russian origins. Polish immigrants are a heterogeneous group with a high percentage of ethnic German resettlers who have an official status as German citizens (for further information on the history of Polish immigration to Germany and the characteristics of Polish immigrants, see [4,9]).

### 1.3. Definition and Measures of Religiosity

Although religion/religiosity is a universal human phenomenon, there is no consensus among researchers regarding a definition for this concept. Religion is a complex and multidimensional construct that is generally associated with specific beliefs, practices, and rituals that are related to a sacred or transcendent (God, higher power, or ultimate truth/reality) and take place within a community but can also be practiced in private [10]. Religion is founded in an established tradition of common beliefs of a group concerning the sacred and is linked to formal institutions such as churches, synagogues, mosques, and temples. Religiosity is considered to be the personal form of the transcendent-related beliefs, experiences, and behaviors [11].

The terms religiosity and spirituality are often used interchangeably in the literature; however, despite some similarities between them, they do not mean the same thing. Spirituality is more difficult to define than religiosity. It is a broader concept than religiosity and is not restricted to religious traditions. Spirituality can be regarded as the individual experience characterized by a quest for meaning in life and the relationship with the sacred or transcendent that can, but does not have to be, connected to formal religious communities and institutions [12]. Another important aspect of religiosity described in the literature is religious coping, whereby religion is used as a coping strategy to manage stressors and critical life events [13].

A wide variety of operationalizations of religion/religiosity exist. More than 125 measurement instruments were identified by Hill and Hood [14]. Several studies have assessed only one aspect of religion using single item scales, for example, asking for the frequency of attendance of religious services or prayer, or the importance of religion in one’s life. However, the reliability and validity of one-item measurement scales is debatable. A multidimensional approach to religion seems to be more adequate.

Two classical theoretical approaches to religion are worth mentioning: the conceptualizations of Allport [15] and Glock [16]. Allport’s concept of intrinsic and extrinsic religiousness has had the greatest impact on the empirical psychology of religion [17]. Glock [16] defined five main dimensions of religion that depict the overall religious life and constitute the frame of reference for empirical research: intellect (interest on religious themes), ideology (beliefs of a religious tradition), religious experience (experiencing the transcendence), and private (e.g., prayer) and public (e.g., participation on public religious services) practices.

The questionnaire applied in the present study to measure religiosity (the Centrality of Religiosity Scale [18]) is a synthesis of these classical approaches. The centrality is conceptualized as the degree of importance or salience of religiosity in the personality of an individual assessed based on the intensity of the five core dimensions of religiosity in one’s life: the more religious a person is, the greater the intensity [19].

### 1.4. Religiosity, (Mental) Health and Health-Related Lifestyle Behaviors

Scientific evidence for the association between religion and mental health is largely based on the Handbook of Religion and Health [12] which is the most comprehensive systematic review ever performed in the field of psychology of religion discussing more than 1200 studies published in the 20th century. A systematic review [20] largely based on this handbook and including additional publications between 2000 and 2005 summarized the main results on the relationship between religiosity and several indicators of mental health and psychological well-being as follows: the majority of studies have shown higher levels of religiosity to be associated with less risk of depression, less suicidal thoughts and behaviors, less alcohol/drug use/abuse, and higher well-being (life satisfaction, happiness, positive affectivity, optimism, hope, self-esteem, sense of meaning in life, internal locus of control, and social support). These beneficial associations remain significant after controlling for important sociodemographic variables and even after adjusting for social support, and they are similar in populations from different countries, religions, ethnic backgrounds, and ages. However, they are more robust among people under stressful circumstances such as those with a medical illness or disability, and among elderly individuals. Also, a systematic evidence-based review [21] including original research published in the top 25% of psychiatry and neurology journals between 1990–2010 found good empirical evidence for the association between religious involvement and less risk of depression, less substance abuse, and less suicide.

Although most of the research on religiosity and health has been conducted in the USA in Christian samples, in recent years, studies from other countries and religions have mostly supported the protective function of religiosity observed in U.S. surveys [20,22].

Despite the growing research interest in the examination of the health status of immigrants as well as the association between religiosity and various indicators of health, the literature on the relationship between religiosity and health-related lifestyle behaviors among immigrants is very scarce, and no investigation has been conducted on the population of Polish immigrants. Most of the studies on this topic have been conducted in the USA in Latino and Asian Indian immigrants. In sum, the scarce literature on the association between religiosity and health-related behaviors in immigrants has consistently shown an inverse relationship between religiosity and tobacco [23,24,25] as well as alcohol consumption or alcohol use disorders ([25,26,27,28]; a positive association between religiosity and physical activity (however, only one study has examined this topic [25]); and mixed results concerning religiosity and being overweight/obese—one survey reported no significant relationship [25] and one [29] demonstrated a positive association (found only for Asian Indians practicing Hinduism or Sikhism, but not for Muslims).

In a recently published systematic review on religion and body weight [30] including 85 studies (49% of the studies included mixed race/ethnicity), a significant association was detected between a higher level of religiosity and a higher body weight in both cross-sectional and prospective investigations—in bivariate analyses but less so in multivariate analyses. Only one of five longitudinal multivariate analyses demonstrated a statistically significant relationship between religiosity and body weight.

### 1.5. Possible Explanations for the Positive Effects of Religiosity on (Mental) Health and Health-Related Lifestyle Behaviors

Several mechanisms have been proposed as explanations for the impact of religion/religiosity on human health, namely, healthy behaviors and lifestyle, cognitive framework, social support, religious practices, spiritual direction, coping, alternative values, and positive emotions [20,31,32]. Religion may improve health by discouraging or even forbidding behaviors that may harm health, such as alcohol (abuse) and drugs, and encouraging people to live in a healthy way (e.g., keeping a day of rest, eating moderately, having peaceful relationships). In addition, religious doctrines that promote attitudes such as forgiveness and empathy, or virtues such as compassion, gratitude, humility, etc., may reduce feelings of anger and hostility and are therefore beneficial for health. Belonging to a religious organization may provide social cohesion and support that promotes health. Religious practices such as prayer and meditation may be forms of relaxation that contribute to reduced stress levels. Religion can provide meaning, orientation, and a sense of control in life as well as helping individuals to cope successfully with stressful and critical life circumstances, such as a severe illness. Furthermore, religious belief systems often emphasize socially critical, alternative values, such as social engagement and humility, and thus, may relieve an individual from the pressure to perform and compete and, consequently, reduce stress. Finally, it is also possible that religion might have a beneficial contribution to health by increasing positive emotions such as happiness and gratitude.

The relationship between religiosity and health has also aroused scientific interest in the field of neuroscience, for example, brain activity during prayer and meditation has been investigated [33]. Also, several physiological mechanisms concerning the central nervous system, neurotransmitters, and the endocrine and immune systems have been proposed to mediate the favorable effects of religiosity on health [34].

Although a large body of studies has generally demonstrated a positive effect of religiosity on various parameters of health, it is important to mention that religion/religiosity (i.e., special religious doctrines and practices) may also have negative effects on human health in some cases, e.g., if a religious group forbids blood transfusions or medications, or leads to social isolation, etc. [31].

Since religion/religiosity is a multidimensional phenomenon, it can be postulated that not the single mechanisms but rather their combination contributes to the favorable effects of religion/religiosity on health.

### 1.6. Study Aims

The homogenous structure of the religious affiliation among the Polish immigrants provides a favorable basis to analyze the impact of (Christian, mostly Catholic) religiosity on health-related lifestyle behaviors.

The aims of the study were:

1. To examine the frequency of health-related lifestyle behaviors (smoking, alcohol consumption, physical activity, and obesity) and categories of religiosity (intrinsic, extrinsic, and no/marginal religiosity) in immigrants of Polish origin in Germany.
2. To investigate the association between religiosity and the four health-related lifestyle behaviors, with adjustment for various important sociodemographic, migration, and health-related characteristics in immigrants of Polish origin.

Based on previous research, we postulated high religiosity to be associated with a lower risk of being a smoker and for alcohol consumption but hypothesized that there is no relationship between religiosity and physical activity or being overweight/obese.

## 2. Materials and Methods

### 2.1. Participants

The recruitment took place in Germany (immigrants of Polish origin) and in Poland (autochthone Poles). The present study focuses only on Polish immigrants. The data obtained have already been used for other publications [9,35,36,37].

In Germany, participants were recruited in centers in the Ruhr area (North Rhine Westphalia, West Germany) where Polish communities had been established, e.g., after Polish language mass, in religious and cultural groups in Polish churches, in non-religious places promoting Polish culture as well as among students from Polish migration backgrounds at the Ruhr University Bochum in Germany. In addition, probands were also recruited by the “snowball” method. This method can be regarded as an alternative sampling strategy if the examined target population is difficult to reach or identify [38], which is the case with immigrants of Polish origin who have German citizenship. Most of the Polish immigrants living in Germany are formally German citizens and thus rank as Germans in statistics; a random sample would, therefore, not include them, whereas data collection within the Polish community made it possible to recruit both immigrants of Polish origin with German as well as immigrants with Polish citizenship.

The inclusion criteria for the study were age of consent (minimum of 18 years), agreement to participate in the study, adequate knowledge of the Polish language due to the fact that the questionnaire was applied only in Polish, residence in the Ruhr area or in neighboring cities, and the status of a Polish immigrant according to the definition applied in epidemiological research in Germany [7], i.e., having immigrated themselves or having at least one parent who immigrated. Therefore, immigrants as well as resettlers were included in the study, regardless of having German citizenship or not.

Four hundred and nine questionnaires were distributed, and 264 were returned (response rate: 56.3%). Two participants were excluded based on the exclusion criteria (residence not in the Ruhr area). Because only five people belonged to the second generation of immigrants (born in Germany), they were excluded from the analyses, so a total sample of *n* = 257 first-generation immigrants were included in the present study.

### 2.2. Procedure and Setting

Data were collected between August 2009 and October 2010 in the abovementioned recruitment centers. Participants completed the self-report questionnaire in Polish language at home.

### 2.3. Ethics Statement

The present study was conducted in accordance with the Declaration of Helsinki, and the protocol was approved by the Ethics Committee of the Medical Faculty of the University of Duisburg-Essen (Project identification code: 11-4723). Written informed consent was obtained from all participants.

### 2.4. Measures

#### 2.4.1. Sociodemographic and Migration-Specific Variables

The following sociodemographic and migration-specific items were assessed: gender, age, marital status, education level, employment status, subjectively perceived income, citizenship, length of residence in Germany, and German language proficiency.

#### 2.4.2. Health-Related Lifestyle Behaviors

Four health-related lifestyle behaviors were measured:

1. The smoking status: current smoker or non-smoker and the number of smoked cigarettes per day: <5, 5–10, 11–20, 21–40, >40.
2. Alcohol consumption: never, seldom, once a month, several times a month, once a week, several times a week, every day.
3. Physical activity per week (sport): never, <1 h, 1–2 h, 2–4 h, >4 h.
4. Body mass index (BMI) category (kg/m^2^): <18.5 (underweight), 18.5–24.9 (normal weight), 25.0–29.9 (overweight), ≥30 (obese). Obesity was defined according to the World Health Organization (WHO) as BMI ≥ 30 kg/m^2^ [39].

#### 2.4.3. Depressive Symptoms

Depressive symptoms were measured by the Beck Depression Inventory (BDI) [40] which consists of 21 items representing the most important symptoms of depression. Scores range between 0 and 63. A total score of 18 points and above indicates clinically relevant depression. In this study, a validated Polish version of the BDI used in clinical studies of the Silesian Medical University [41] was employed, which obtained a Cronbach’s alpha score of 0.90 in the present sample.

#### 2.4.4. Anxiety Symptoms

Anxiety symptoms were assessed by the Beck Anxiety Inventory (BAI) [42] which comprises 21 items (13 related to somatic anxiety symptoms, 5 to cognitive aspects of anxiety, and 3 items measure both cognitive and somatic symptoms). Scores between 0 and 63 can be achieved. In the present sample, the Cronbach’s alpha score of the validated Polish version [41] was 0.91. A cut-off-value of 26 indicates clinically relevant anxiety.

#### 2.4.5. Somatic Symptoms

The brief form of the Giessen Subjective Complaints List (GBB-24) [43], which consists of 24 items, was employed to assess somatic symptoms. Scores range between 0 and 96. Four subscales (“exhaustion”, “stomach complaints”, “pain in the extremities” and “heart complaints”) and a total score can be calculated (“symptom pressure”). In the present study, a validated Polish version developed by the research group of the Department of Medical Psychology and Medical Sociology at the University of Leipzig [44] was used. The Cronbach’s alpha score in the examined sample was 0.93.

#### 2.4.6. Perceived Discrimination

Subjectively perceived discrimination was assessed by means of four self-constructed items on a 1–5 Likert scale (higher scores indicating higher perceived discrimination) concerning the categories “neighborhood”, “shopping”, “administrative office”, and “working life” (see [36]). A total score was created as an index for the discrimination (there was a Cronbach’s alpha score of 0.78. in the present study).

#### 2.4.7. Sense of Coherence

The Sense of Coherence Scale (SOC-29) is a self-report questionnaire consisting of 29 items on a seven-point Likert scale designed to assess the extent of coherence [45] which represents the basic human orientation consisting of three components: comprehensibility, manageability, and meaningfulness. A higher score indicates a higher SOC. In the present study, an adapted Polish version of the SOC-29 was applied that has been proven to have high internal consistency with Cronbach’s alpha coefficients ranging from 0.81 to 0.91 for the global scale and the subscales [46]. In the present study, the Cronbach’s alpha score for the validated Polish version was 0.92.

#### 2.4.8. Religiosity

The Centrality of Religiosity Scale (CRS) [18] was applied to assess religiosity. It comprises 15 items that categorize the respondents into not/marginally religious (0–15 points), extrinsically (16–44), and intrinsically religious (45–60). The CRS has been employed in more than 100 studies in 25 countries and has excellent psychometric properties (Cronbach’s alpha = 0.92–0.96) [19]. The Cronbach’s alpha score of the validated Polish version [47] in the study sample was 0.96.

### 2.5. Statistical Analysis

Data analyses were conducted with SPSS V. 21 (IBM, Armonk, NY, USA). Missing values in the questionnaires were replaced with the expectation-maximization algorithm (max. 20% missing data per questionnaire was accepted, otherwise the case was excluded from the analysis). The following descriptive statistics were computed to profile the sociodemographic and migration-specific sample characteristics and the lifestyle behaviors: means, standard deviations, ranges, and frequencies. To test for differences between women and men for categorical data, parametric tests (*χ*^2^-tests or Fisher’s exact test) were performed and for continuous data, t-tests for independent samples were computed. Pearson’s correlation analyses among the main psychological variables were calculated prior to estimating regressions to test multicollinearity (by inspection of a pairwise correlation matrix). If the correlation coefficients were higher than 0.70 [48], one of the variables was excluded from the logistic regression models. Multicollinearity was also checked by calculating the variance inflation factors. Binary logistic regression analyses were conducted with the enter method to examine the influence of sociodemographic, migration- and health-related variables and religiosity on smoking, alcohol consumption, physical activity, and being overweight/obese. The three non-categorical lifestyle behaviors and the religiosity were dichotomized: frequency of alcohol (no/seldom consumption vs. all other categories), physical activity (no activity/<1 h vs. all other categories), BMI (under/normal weight vs. overweight/obesity) and religiosity (intrinsic vs. extrinsic/marginal). For the predictors, we report odds ratios (ORs) and 95% confidence intervals (CIs). A level of significance of *p* < 0.05 was predetermined.

## 3. Results

### 3.1. Sociodemographic and Migration-Specific Data

In Table 1, the main sociodemographic and migration-specific characteristics are presented for the total sample as well as for women and men of Polish origin living in Germany. Approximately two-thirds (64.6%) of the participants were women. Immigrant women were significantly younger than immigrant men (*p* = 0.004), were more likely to be unemployed (*p* = 0.001), to have a (very) low income (*p* = 0.001) but a higher education (*p* < 0.001), to have lived for a shorter period of time in Germany than the men (*p* = 0.027), and they more frequently had Polish citizenship (*p* = 0.001).

### 3.2. Frequency and Gender-Specific Differences Regarding Health-Related Lifestyle Behaviors and Religiosity Levels

Table 2 shows the frequency of smoking, alcohol consumption, physical activity, BMI, and religious categories in the examined sample. Immigrant men were less frequently smokers (*p* = 0.022) but more frequently were overweight/obese (*p* < 0.001) than immigrant women. Regarding the number of smoked cigarettes per day, the frequency of alcohol consumption, and the physical activity level as well as the level of religiosity, no significant gender-specific differences were found.

### 3.3. Correlations between Potential Psychological Predictors

To avoid multicollinearity, bivariate correlations were computed among the main psychological variables. The correlation coefficients are presented in Table 3. Correlation coefficients higher than 0.70 indicate potential multicollinearity [48]. In such cases, one of the two variables was not included in the logistic regression models. Two correlations showed a correlation coefficient >0.70: between depressive symptoms and sense of coherence (r = −0.74) and between anxiety and somatic symptoms (r = 0.73). Due to this fact, sense of coherence and somatic symptoms were excluded from the logistic regressions.

### 3.4. Predictors of Health-Related Lifestyle Behaviors

The odds ratios for the total sample are depicted in Table 4. After adjusting for various sociodemographic, migration, and health-related characteristics, intrinsic religiosity was significantly associated with a lower risk of being a smoker (OR = 0.34, CI = 0.15–0.76, *p* = 0.009) and also a lower risk of alcohol consumption (OR = 0.33, CI = 0.15–0.71, *p* = 0.005) in comparison with extrinsic/marginal/non-religiosity. No relationship was found between religiosity and physical activity. Highly religious immigrants were more likely to be overweight/obese than extrinsically, marginally, and non-religious immigrants (OR = 2.53, CI = 1.15–5.56, *p* = 0.021).

Other significant predictors for smoking were gender and depression: women and more depressed individuals had a higher risk of being a smoker. Concerning alcohol consumption, younger age, masculine gender, lower discrimination levels, and higher anxiety were further significant predictors. For physical activity, significant tendencies were only detected for gender (*p* = 0.089) and age (*p* = 0.09): older individuals and women were more likely to be physically inactive. In the full model for overweight/obesity, gender, depression, and age were also significant predictors: masculine gender, older age and a higher depression level were risk factors for being overweight/obese. The explanation of variance for the full model was 13.7% for smoking, 22.2% for alcohol consumption, 15.0% for physical activity, and 44.0% for overweight/obesity, respectively.

## 4. Discussion

To the best of our knowledge, this is the first study to examine the association between religiosity and various indicators of health-related behaviors in immigrants of Polish origin.

The main result of the present investigation is the significantly lower risk of being a current smoker and for drinking alcohol in highly religious individuals in comparison with less religious or non-religious people. This result is consistent with findings from previous studies with immigrant samples ([23,24,25,26,27,28] and with the results of the Polish General Social Survey (*n* = 1526) [49] and the Survey of Health, Ageing, and Retirement in Europe (*n* = 16,557) ([50]. Several possible explanations can be provided for the protective role of religiosity against substance consumption. It can be postulated, in accordance with Huber [18], that highly religious people have internalized the moral norms, values, and believes of their religion. Thus, they are more likely to also follow lifestyle-related religious prohibitions with detrimental effects to their health such as smoking and drinking alcohol. Furthermore, it can be postulated that the sense of meaning and control in life provided by a religion as well as social support from the religious community can contribute to the successful management of problems and adverse life circumstances in high religious individuals, so they do not use nicotine or alcohol as self-medications for emotion regulation. A further possible explanation is a stress-reducing effect of religious practices such as prayer, meditation, worships, etc., which is supported by several studies [51]. Thus, highly religious involved people who regularly perform these practices can benefit from them as a “by-product”. In addition, the internalized attitudes of highly religious people, such as forgiveness or compassion, are related to decreased unhealthy feelings of anger and hostility and may also have a stress-reducing impact [52]. Religiosity may also influence the personality towards self-control and emotional stability/ positive affectivity [20]. Finally, it could be possible that the positive emotions associated with religiosity make artificial “mood lifters” such as smoking and alcohol superfluous.

Our results suggest that a high level of religiosity may not have the same beneficial effects on physical activity and on weight as it does on smoking and drinking. A possible reason for this is that the Catholic religion has a more negative attitude towards substance abuse than towards inactivity and being overweight. In particular, alcohol abuse is regularly denounced, and abstinence is promoted by the Catholic Church. Different religious programs are promoted by priests, such as total abstinence in August for adults or total abstinence for adolescences for a year. Such large engagement for other (un)healthy lifestyle behaviors is not realized.

Highly religious people showed an increased risk of being overweight/obese. This result was also demonstrated in a study with immigrants from the USA [29]. One explanation could be that high religiosity is associated with a lower risk of being a smoker. As a result, highly religious people cannot benefit from nicotine as an appetite suppressant. According to the review on religion and bodyweight mentioned above [30], the relationship between religiosity and higher body weight is still unclear. Other determinants not examined in this study could have mediated the association between religiosity and being overweight/obese and should be explored in future investigations to allow a better understanding of the underlying mechanisms.

An important finding of our study is the high smoking rate (35.5%) in women. Also, the regression model provided support for the conclusion that immigrant women of Polish origin are a risk group for cigarette smoking. This gender difference found in our study does not match trends, as (immigrant) men are more likely to smoke than (immigrant) women [53,54,55]. A possible explanation for the high rate of tobacco use in immigrant women is that tobacco consumption may be used as a strategy to deal with (acculturative) stress, various burdens, and multiple experiences of discrimination (as women, immigrants, and frequently low-paid workers). In addition, the high probability of women to be smokers may be partially explained by the level of acculturation. An increased smoking prevalence was observed to be associated with increased acculturation among Hispanic women and Asian women in the USA [56,57]. In our sample, more than 70% of the immigrant women assessed their own German language proficiency (one of the best indicators of acculturation) as being at least as good and thus can be regarded as highly acculturated and hence, more strongly influenced by practices of the host society than less acculturated individuals [53]. As a result, their smoking patterns may converge to those of the host society, as has been shown for resettlers from the Former Soviet Union in Germany [58]. This could also be partially postulated for the women in the present study: a lower smoking prevalence was observed for women in Poland (23% [54]) than those in our study (35.5%). However, among the immigrant women, a higher proportion of smokers was found than among women in Germany (27% [55]). Thus, other influencing factors on the high smoking rate and the increased risk of being a smoker in immigrant women as compared with the men can be assumed and these should be investigated in future research.

Another important gender-related difference in this study was the significantly higher proportion of overweight/obese men than women. The male gender was also found to be a risk factor for being overweight/obese in the regression analysis. The higher probability of being overweight/obese among immigrant men is in line with the results of some prior studies [29,59], while other studies reported a higher prevalence in immigrated women in relation to men [60]. One plausible explanation for the increased odds ratio of overweight/obese men in our investigation may be the high prevalence of smokers among women. Smoking has been shown to be negatively linked to the probability of obesity in different immigrant groups in the USA [61]. The high odds for being overweight/obese in men could also be explained by their lower education level as compared with the women. The protective function of high educational attendance has been demonstrated in immigrants [61] as well as in populations of different countries [62]. It may contribute to a healthier lifestyle in the form of, e.g., healthier dietary practices.

It is notable that a large proportion of our sample are physically inactive and are also overweight/obese. Almost two-thirds of the immigrants do not participate in any sport activity or participate for less than 1 h per week. According to the WHO, at least 150 min of moderate intensity physical activity per week is recommended to receive benefits on health [63]. However, less than 20% of the examined immigrants stated that they follow this recommendation. In the multivariable analysis, there was a tendency for higher age and female gender to be associated with an increased risk of physical inactivity. The poor health of the older adults could prevent them from sport participation [64]. Women may be involved in less sport activities because of their multiple roles, stressors, and responsibilities meaning they have less time for leisure activities.

In the present study, high levels of depressive or anxiety symptoms were associated with a higher risk of smoking, alcohol consumption, and being overweight/obese. This finding is in line with empirical evidence supporting the association between worse lifestyles and depressive and anxiety symptoms [65]. It can be postulated that people with mental health problems use smoking, drinking, and/or eating as dysfunctional “coping strategies” against negative mood states.

### Strengths and Limitations

The primary strength of the present study is that we applied a multidimensional, well conceptualized, reliable, and valid measurement of religiosity in a relatively large, religiously homogenous population of immigrants of Polish origin. Furthermore, we were able to include individuals who are difficult to identify for surveys (immigrants of Polish origin with German citizenship) and people who are often excluded from participation in studies in Germany because of insufficient language competencies (immigrants of Polish origin with insufficient German language proficiency). In addition, we included many potential moderator factors of religiosity in the regression models; thus, we were able to control for several confounding variables when examining the impact of religiosity on the indicators of health-related lifestyle behaviors.

The results of our study should be interpreted in light of some limitations. The cross-sectional design of the study did not allow causal conclusions regarding the influence of the variables examined on the health-related behavior to be drawn. Prospective studies are therefore needed to investigate the associations found in the study and to detect underlying mechanisms. Another limitation was the low response rate (56.3%), which may have led to a selection bias, possibly with healthier immigrants being included in the sample. The “snowball method” could also have biased the results. Thus, the findings should be viewed as exploratory and cannot be generalized to the population of all immigrants of Polish origin living in Germany or to other ethnic minorities. Moreover, the non-objective, self-reported estimates of the four health-related lifestyle behaviors may also have been a source of bias because (some) respondents may not have reported the real data due to social desirability or shame (especially for body weight or the frequency of alcohol consumption). Studies indicate that women may underestimate their body weight, while men may overestimate their height [66]. Finally, the item used to measure the physical activity (sport) is not precise and thus is of questionable validity. Valid measures such as MVPA (moderate to vigorous physical activity) should be applied in future research to ensure valid evaluation of physical activity levels. Future studies should examine the association between religiosity and health-related behavior in representative samples that also include second generation immigrants, taking into account other important psychological (e.g., self-esteem), psychosocial (e.g., social support), lifestyle (e.g., dietary habits) or acculturation-related (e.g., acculturative stress) variables that were not examined in this study, and separate analyses should be performed for men and women.

## 5. Conclusions

Due to the limitations in the study design (cross-sectional, snowball method) and the low response rate, the findings should be interpreted with caution. Several conclusions can be drawn from the results that provide important implications for public health. First, our findings indicate that high religiosity can be regarded as a protective factor against tobacco and alcohol consumption in immigrants of Polish origin. This should be considered in prevention programs. Secondly, immigrant Polish women present a risk group for smoking and should be targeted in preventive gender and immigration-specific anti-smoking programs. Thirdly, highly religious people and men are at a higher risk of being overweight/obese. Religious leaders and local religious communities should therefore be involved in preventive programs to promote not only abstinence from smoking and drinking but also a healthy lifestyle with physical activity and a healthy diet. Fourth, depressive and anxiety symptoms were shown to increase the likelihood for smoking, drinking, and being overweight/obese. Psychological support should be offered to immigrants with mental health problems to avoid dysfunctional coping with cigarettes or alcohol. Finally, religiosity can still be regarded as the “forgotten factor” in the research of (mental) health [21], so it remains a desideratum for researchers and healthcare providers to take this variable into account in their studies and clinical practice.

## Figures and Tables

**Table 1:** Sociodemographic, socio-economic, and immigrant-specific sample characteristics, and differences by gender.

Variables		Total Sample (*n* = 257)	Women (*n* = 166)	Men (*n* = 91)	*p*-Value
Age	mean	42.8	41.0	46.2	0.004 ^a^
	SD *	14.0	13.6	14.0	
	range	18-84	18-76	20-84	
Marital status	married	155 (60.3%)	94 (56.6%)	61 (67.0%)	0.174 ^b^
	single	68 (26.5%)	50 (30.1%)	18 (19.8%)	
	divorced/widowed	33 (12.8%)	22 (13.3%)	11 (12.1%)	
	no data	1 (0.4%)	0	1 (1.1%)	
Education	low	76 (29.6%)	41 (24.7%)	35 (38.5%)	<0.001 ^b^
	middle (university entrance diploma)	114 (44.4%)	69 (41.6%)	45 (49.5%)	
	high (university degree)	64 (24.9%)	54 (32.5%)	10 (11.0%)	
	no data	3 (1.2%)	2 (1.2%)	1 (1.1%)	
Employment status	employed	149 (58.0%)	84 (50.6%)	65 (71.4%)	0.001 ^b^
	unemployed (housewife/jobless/pensioner/student)	105 (40.9%)	80 (48.2%)	25 (27.5%)	
	no data	3 (1.2%)	2 (1.2%)	1 (1.1%)	
Subjectively perceived income	no income/very low/low	115 (44.7%)	88 (53.0%)	27 (29.7%)	0.001 ^b^
	middle	131 (51.0%)	71 (42.8%)	60 (65.9%)	
	high/very high	9 (3.5%)	6 (3.6%)	3 (3.3%)	
	no data	2 (0.8%)	1 (0.6%)	1 (1.1%)	
Citizenship	German	100 (38.9%)	59 (35.5%)	41 (45.1%)	0.011 ^b^
	Polish	76 (29.6%)	60 (36.1%)	16 (17.6%)	
	German and Polish	75 (29.2%)	45 (27.1%)	30 (33.0%)	
	no data	6 (2.3%)	2 (1.2%)	4 (4.4%)	
Length of residence in Germany	mean	18.0	17.2	19.3	0.027 ^a^
	SD *	7.6	8.2	6.4	
	range	<1–53	<1–53	<1–29	
Language proficiency	excellent	37 (14.4%)	27 (16.3%)	10 (11.0%)	0.382 ^b^
	very good	38 (14.8%)	28 (16.9%)	10 (11.0%)	
	good	96 (37.4%)	62 (37.3%)	34 (37.4%)	
	moderate	65 (25.3%)	37 (22.3%)	28 (30.8%)	
	little	11 (4.3%)	7 (4.2%)	4 (4.4%)	
	no data	10 (3.9%)	5 (3.0%)	5 (5.5%)	

* SD = Standard deviation; ^a^ t-test; ^b^ *χ*^2^-test.

**Table 2:** Frequency of cigarette smoking, alcohol consumption, physical activity, body mass index (BMI), and religiosity categories in immigrants of Polish origin in Germany.

Variables	Total (*n* = 257)	Women (*n* = 166)	Men (*n* = 91)	*p*-Value
Smoking status, *n* (%)				0.022 ^b^
Non-smoker	177 (68.9)	106 (63.9)	71 (78.0)	
Current smoker	79 (30.7)	59 (35.5)	20 (22.0)	
No data	1 (0.4)	1 (0.6)	-	
Smoked cigarettes per day *, *n* (%)				0.341 ^c^
<5	12 (15.2)	10 (16.9)	2 (10.0)	
5–10	25 (31.6)	21 (35.6)	4 (20.0)	
11–20	32 (40.5)	22 (37.3)	10 (50.0)	
21–40	10 (12.7)	6 (10.2)	4 (20.0)	
Alcohol consumption, *n* (%)				0.278 ^c^
Never	14 (5.4)	9 (5.4)	5 (5.5)	
Seldom	143 (55.6)	100 (60.2)	43 (47.3)	
Once a month	10 (3.9)	6 (3.6)	4 (4.4)	
Several times a month	46 (17.9)	25 (15.1)	21 (23.1)	
Once a week	27 (10.5)	18 (10.8)	9 (9.9)	
Several times a week	11 (4.3)	6 (3.6)	5 (5.5)	
Everyday	4 (1.6)	1 (0.6)	3 (3.3)	
No data	2 (0.8)	1 (0.6)	1 (1.1)	
Physical activity per week, *n* (%)				0.423 ^b^
None	100 (38.9)	68 (41.0)	32 (35.2)	
<1 h	63 (24.5)	40 (24.1)	23 (25.3)	
1–2 h	42 (16.3)	29 (17.5)	13 (14.3)	
2–4 h	28 (10.9)	17 (10.2)	11 (12.1)	
>4 h	23 (8.9)	11 (6.6)	12 (13.2)	
No data	1 (0.4)	1 (0.6)	-	
BMI categories (kg/m^2^), *n* (%)				<0.001 ^c^
<18.5 (underweight)	5 (1.9)	5 (3.0)	0	
18.5–24.9 (normal weight)	125 (48.6)	104 (62.7)	21 (23.1)	
25.0–29.9 (overweight)	97 (37.7)	44 (26.5)	53 (58.2)	
≥30 (obesity)	24 (9.3)	8 (4.8)	16 (17.6)	
No data	6 (2.3)	5 (3.0)	1 (1.1)	
BMI, Mean (SD)	24.94 (3.90)	23.69 (3.60)	27.19 (3.40)	<0.001
Religiosity, *n* (%)				0.646 ^b^
Intrinsically religious	71 (27.6)	48 (28.9)	23 (25.3)	
Extrinsically religious	141 (54.9)	88 (53.0)	53 (58.2)	
Not/marginally religious	39 (15.2)	27 (16.3)	12 (13.2)	
No data	6 (2.3)	3 (1.8)	3 (3.3)	
Mean (SD)	34.20 (15.0)	33.94 (15.44)	34.69 (14.24)	0.709 ^a^

* *n* = 79 current smokers (59 women and 20 men); SD = Standard deviation; ^a^ t-test; ^b^ *χ*^2^-test; ^c^ Fisher´s exact test.

**Table 3:** Pearson’s correlation coefficients between central study variables in immigrants of Polish origin in Germany.

Variables	1	2	3	4	5	6
1. Depressive symptoms (BDI)	1	0.61 ***	0.67 ***	0.33 ***	−0.74 ***	0.02
2. Anxiety symptoms (BAI)	-	1	0.73 ***	0.35 ***	−0.59 ***	0.11
3. Somatic symptoms (GBB-24)	-	-	1	0.37 ***	−0.57 ***	0.18 **
4. Perceived discrimination (self-constructed items)	-	-	-	1	−0.35 ***	0.04
5. Sense of Coherence (SOC-29)	-	-	-	-	1	0.05
6. Religiosity (CRS)	-	-	-	-	-	1

** *p* ≤ 0.01, *** *p* ≤ 0.001; BDI: Beck Depression Inventory, BAI: Beck Anxiety Inventory, GBB-24: Giessen Subjective Complaints List, SOC-29: Sense of Coherence Scale, CRS: Centrality of Religiosity Scale.

**Table 4:** Odds Ratios (OR) with 95% confidence intervals (CI) for smoking, alcohol consumption, physical activity, and overweight/obesity in immigrants of Polish origin.

Independent Variables	Smoking		Alcohol		Physical Activity		Weight/Obesity	
	Predictors *: OR [95% CI], *p*-Value	EV ^#^	Predictors: OR [95% CI], *p*-Value	EV	Predictors: OR [95% CI], *p*-Value	EV	Predictors: OR [95% CI], *p*-Value	EV
Model 1: Socio-demo-graphic variables	**1. gender: 1.99 [1.06–3.74], *p* = 0.033** 2. age 0.99 [0.97–1.01], *p* = 0.381 3. education: 0.94 [0.50–1.76], *p* = 0.848 4. employment: 1.16 [0.63–2.14], *p* = 0.637 5. income: 0.96 [0.52–1.79], *p* = 0.900	3.6	**1. gender: 0.44 [0.24–0.81], *p* = 0.008** **2. age: 0.95 [0.93–0.98], *p* < 0.001** 3. education: 1.08 [0.59–2.01], *p* = 0.797 4. employment: 1.43 [0.76–2.72], *p* = 0.271 5. income: 0.98 [0.53–1.83], *p* = 0.956	13.7	1. gender: 0.56 [0.30–1.02], *p* = 0.057 **2. age: 0.96 [0.94–0.98], *p* < 0.001** 3. education: 1.65 [0.92–2.99], *p* = 0.095 4. employment 0.76 [0.42–1.40], *p* = 0.383 5. income 1.60 [0.87–2.95], *p* = 0.132	12.4	**1. gender: 0.15 [0.08–0.30], *p* < 0.001** **2. age: 1.06 [1.04–1.09], *p* < 0.001** 3. education: 0.74 [0.38–1.45], *p* = 0.382 4. employment: 1.07 [0.55–2.07], *p* = 0.846 5. income: 1.14 [0.59–2.22], *p* = 0.698	37.1
Model 2: Socio-demo-graphic + migration- specific variables + discrimination	**1. gender: 2.28 [1.17–4.47], *p* = 0.016** 2. age: 0.97 [0.94–1.0], *p* = 0.082 3. education: 0.86 [0.45–1.67], *p* = 0.660 4. employment: 1.21 [0.61–2.41], *p* = 0.585 5. income: 1.05 [0.53–2.07], *p* = 0.892 6. length of residence: 1.05 [0.99–1.1], *p* = 0.112 7. language: 0.83 [0.57–1.22], *p* = 0.350 8. discrimination:1.05 [0.95–1.16], *p* = 0.374	6.7	**1. gender: 0.52 [0.28–0.98], *p* = 0.044** **2. age: 0.94 [0.91–0.98], *p* = 0.001** 3. education: 1.10 [0.57–2.13], *p* = 0.772 4. employment: 1.73 [0.84–3.54], *p* = 0.137 5. income: 0.86 [0.43–1.70], *p* = 0.665 6. length of residence: 1.05 [0.99–1.10], *p* = 0.087 7. language: 0.99 [0.68–1.44], *p* = 0.967 8. discrimination: 0.92 [0.83–1.03], *p* = 0.131	15.6	1. gender: 0.60 [0.31–1.14], *p* = 0.115 2. age: 0.97 [0.94–1.0], *p* = 0.072 3. education: 1.77 [0.94–3.31], *p* = 0.075 4. employment: 0.82 [0.42–1.63], *p* = 0.575 5. income: 1.42 [0.72–2.79], *p* = 0.306 6. length of residence: 1.01 [0.96–1.07], *p* = 0.710 7. language: 1.34 [0.93–1.95], *p* = 0.119 8. discrimination: 1.01 [0.92–1.12], *p* = 0.787	14.0	**1. gender: 0.14 [0.07–0.28], *p* < 0.001** **2. age: 1.05 [1.01–1.09], *p* = 0.010** 3. education: 0.70 [0.34–1.43], *p* = 0.330 4. employment: 0.99 [0.47–2.08], *p* = 0.970 5. income: 1.29 [0.62–2.71], *p* = 0.500 6. length of residence: 1.02 [0.96–1.08], *p* = 0.512 7. language: 0.91 [0.60–1.38], *p* = 0.653 8. discrimination: 1.0 [0.89–1.12], *p* = 0.962	38.3
Model 3: Socio-demo-graphic + migration- specific variables + discrimination + health-related variables	**1. gender: 2.3 [1.14–4.64], *p* = 0.019** 2. age: 0.97 [0.94–1.01], *p* = 0.091 3. education: 1.03 [0.52–2.06], *p* = 0.933 4. employment: 1.26 [0.63–2.55], *p* = 0.515 5. income: 1.13 [0.56–2.26], *p* = 0.738 6. length of residence: 1.05 [0.99–1.11], *p* = 0.106 7. language: 0.84 [0.57–1.24], *p* = 0.386 8. discrimination: 1.03 [0.92–1.15], *p* = 0.621 **9. depressive symptoms: 1.06 [1.01–1.12], *p* = 0.025** 10. anxiety symptoms: 0.98 [0.94–1.02], *p* = 0.241	9.1	**1. gender: 0.44 [0.23–0.87], *p* = 0.017** **2. age: 0.94 [0.91–0.98], *p* = 0.001** 3. education: 1.03 [0.52–2.03], *p* = 0.929 4. employment: 1.81 [0.87–3.76], *p* = 0.110 5. income: 0.91 [0.45–1.83], *p* = 0.793 6. length of residence: 1.05 [0.99–1.10], *p* = 0.095 7. language: 1.01 [0.70–1.47], *p* = 0.955 8. discrimination: 0.89 [0.79–1.0], *p* = 0.053 9. depressive symptoms: 0.99 [0.94–1.04], *p* = 0.666 10. anxiety symptoms: 1.04 [1.0–1.08], *p* = 0.068	17.8	1. gender: 0.54 [0.28–1.06], *p* = 0.074 2. age: 0.97 [0.94–1.01], *p* = 0.110 3. education: 1.61 [0.84–3.08], *p* = 0.155 4. employment: 0.84 [0.42–1.69], *p* = 0.626 5. income: 1.33 [ 0.66–2.65], *p* = 0.425 6. length of residence: 1.01 [0.95–1.06], *p* = 0.836 7. language: 1.36 [0.94–1.97], *p* = 0.108 8. discrimination: 1.01 [0.90–1.13], *p* = 0.867 9. depressive symptoms: 0.96 [0.91–1.01], *p* = 0.133 10. anxiety symptoms: 1.03 [0.99–1.07], *p* = 0.213	15.1	**1. gender: 0.13 [0.06–0.26], *p* < 0.001** **2. age: 1.05 [1.01–1.09], *p* = 0.013** 3. education: 0.83 [0.39–1.75], *p* = 0.618 4. employment: 1.03 [0.48–2.21], *p* = 0.946 5. income: 1.51 [0.71–3.24], *p* = 0.288 6. length of residence: 1.02 [0.96–1.09], *p* = 0.460 7. language: 0.92 [0.60–1.40], *p* = 0.682 8. discrimination: 0.97 [0.86–1.10], *p* = 0.656 **9. depressive symptoms: 1.08 [1.02–1.15], *p* = 0.013** 10. anxiety symptoms: 0.98 [0.94–1.03], *p* = 0.46	40.9
Model 4: Socio-demographic + migration-specific variables + discrimination + health-related variables + religiosity	**1. gender: 2.57 [1.25–5.29], *p* = 0.01** 2. age: 0.98 [0.95–1.02], *p* = 0.398 3. education: 1.07 [0.53–2.16], *p* = 0.849 4. employment: 1.22 [0.59–2.51], *p* = 0.599 5. income: 1.14 [0.55–2.35], *p* = 0.721 6. length of residence: 1.03 [0.97–1.09], *p* = 0.294 7. language: 0.83 [0.57–1.23], *p* = 0.363 8. discrimination: 1.03 [0.91–1.15], *p* = 0.682 **9. depressive symptoms: 1.05 [1.0–1.11], *p* = 0.049** 10. anxiety symptoms: 0.98 [0.95–1.02], *p* = 0.418 **11. religiosity: 0.34 [0.15–0.76], *p* = 0.009**	13.7	**1. gender: 0.43 [0.22–0.86], *p* = 0.017** **2. age: 0.95 [0.92–0.99], *p* = 0.012** 3. education: 1.11 [0.56–2.21], *p* = 0.772 4. employment: 1.83 [0.86–3.91], *p* = 0.117 5. income: 0.89 [0.43–1.83], *p* = 0.746 6. length of residence: 1.03 [0.98–1.09], *p* = 0.247 7. language: 1.0 [0.68–1.46], *p* = 0.985 **8. discrimination: 0.88 [0.78–1.0], *p* = 0.042** 9. depressive symptoms: 0.98 [0.93–1.03], *p* = 0.430 **10. anxiety symptoms: 1.05 [1.0–1.09], *p* = 0.032** **11. religiosity: 0.33 [0.15–0.71], *p* = 0.005**	22.2	1. gender: 0.56 [0.28–1.09], *p* = 0.089 2. age: 0.97 [0.94–1.01], *p* = 0.090 3. education: 1.57 [0.82–3.02], *p* = 0.176 4. employment: 0.84 [0.42–1.70], *p* = 0.627 5. income: 1.35 [0.67–2.74], *p* = 0.405 6. length of residence: 1.01 [0.96–1.06], *p* = 0.769 7. language: 1.35 [0.93–1.96], *p* = 0.115 8. discrimination: 1.01 [0.90–1.14], *p* = 0.820 9. depressive symptoms: 0.96 [0.91–1.01], *p* = 0.153 10. anxiety symptoms: 1.03 [0.98–1.07], *p* = 0.234 11. religiosity: 1.23 [0.61–2.47], *p* = 0.561	15.0	**1. gender: 0.11 [0.05–0.24], *p* < 0.001** **2. age: 1.04 [1.0–1.08], *p* = 0.047** 3. education: 0.85 [0.39–1.82], *p* = 0.668 4. employment: 1.25 [0.56–2.76], *p* = 0.587 5. income: 1.18 [0.54–2.60], *p* = 0.678 6. length of residence: 1.04 [0.97–1.10], *p* = 0.276 7. language: 0.91 [0.59–1.41], *p* = 0.672 8. discrimination: 0.97 [0.85–1.11], *p* = 0.668 **9. depressive symptoms: 1.09 [1.02–1.15], *p* = 0.01** 10. anxiety symptoms: 0.98 [0.93–1.02], *p* = 0.331 **11. religiosity: 2.53 [1.15–5.56], *p* = 0.02**	44.0

Model 1: adjusted for age, gender (men (ref.) vs. women), education (low (ref.) vs. middle/high), employment status (unemployed (ref.) vs. employed), subjectively perceived income (no income/very low/low (ref.) vs. middle/high/very high); Model 2: additionally adjusted for length of residence in Germany, language proficiency (higher scores = higher proficiency); discrimination (higher scores = higher discrimination); Model 3: additionally adjusted for depressive and anxiety symptoms; Model 4: additionally adjusted for religiosity (extrinsic/marginal/non-religious (ref.) vs. intrinsic); * significant predictors are marked in bold; ^#^ EV: explanation of variance (%).
